# A Training System for Human Standing Stability Using Virtual Viscosity Fields

**DOI:** 10.3390/s26061985

**Published:** 2026-03-22

**Authors:** Hayato Mikami, Keisuke Shima, Tianyi Wang, Haruto Kai, Koji Shimatani

**Affiliations:** 1 Graduate School of Environment and Information Sciences, Yokohama National University, Yokohama 240-8501, Japan; 2 Faculty of Environment and Information Sciences, Yokohama National University, Yokohama 240-8501, Japan; wang-tianyi-yf@ynu.ac.jp; 3 Faculty of Health and Welfare, Prefectural University of Hiroshima, Mihara 723-0053, Japan; shimatani@pu-hiroshima.ac.jp

**Keywords:** limits of stability (LOS), fall prevention, postural stability training

## Abstract

Enhancement of postural stability in standing is essential for fall prevention in the context of demographic aging. Against such a background, this study proposes a personalized training system based on individual limits of stability (LOS) for a human standing state. The system evaluates LOS in eight directions using center-of-mass (COM) and center-of-pressure (COP) measurement devices and provides game-based feedback, then promotes balance within the relevant LOS parameters. Loading is individualized by applying greater force to virtual objects as the COP approaches the LOS determined for each subject. Experiments with 32 younger and 19 mature subjects produced evaluations for postural stability index (IPS), LOS area, and COP sway. The results revealed two distinct response patterns: LOS expansion and sway reduction, both observed across younger and mature cohorts. These findings suggest that individualized LOS-based training can be applied to improve standing stability with two distinct strategies. These preliminary findings suggest that individualized LOS-based training is associated with changes in standing stability through two distinct response patterns.

## 1. Introduction

Japan’s demographic aging rate has followed an increasing trend in recent years. In 2023, the proportion of people aged 65 and over relative to the total population stood at a relatively high 29.1% [1]. In this context, falls among the elderly represent a significant public health concern, contributing substantially to emergency hospital admission numbers [2], and extensive research has been undertaken to prevent such accidents. Various prevention strategies have been explored, including improved walker designs [3], hip protectors for fracture prevention [4] and intervention programs tailored to individual risk factors [5]. While these approaches are effective in reducing fall risk, enhancing elderly individuals’ intrinsic balance and postural control capabilities remains critically important. Against this background, evaluation and training for standing stability are significant considerations.

**Figure 1:**
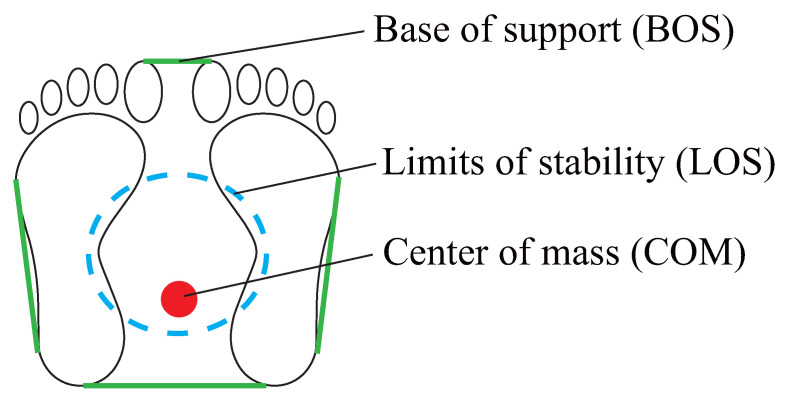
Elements of postural stability.

Figure 1 illustrates the key elements involved in standing postural stability. From a kinematic perspective, stability is highly correlated to relationships on the horizontal plane between the body’s limits of stability (LOS) and center of mass (COM) when the base of support (BOS) is fixed. LOS are defined as the maximum range of COM movement possible within the BOS parameters [6], and maximum COM ranges are simply approximated as those from center of pressure (COP) evaluation. As LOS are known to diminish with age [7], leading to increased postural instability, various forms of training to improve these limits have been covered in previous research. For instance, Hasegawa et al. considered intervention involving static standing maintenance on an inclined platform among elderly patients with Parkinson’s disease who were at high risk of falls, with results showing LOS anterior expansion [8]. Vassilis et al. conducted dynamic training for elderly individuals and reported LOS expansion from weight-shifting exercises accompanied by visual guidance [9]. More recently, Vos et al. demonstrated the training potential of visual feedback for improving dynamic postural stability [10]. Plaiwan et al. considered training with individual LOS [11]. In the study, subjects aged 50 and over repeatedly performed training exercises involving maintenance of 75% of their own LOS for 3 s, with results showing a tendency toward cognitive improvement; however, a reduction in maximum forward reach distance was also observed. Various other technological approaches have also been developed, but tend to be large-scale and costly, requiring specialized equipment. These factors present obstacles to implementation, and may hinder broader clinical adoption.

This paper presents an effective methodology using a simple game system to provide personalized improvement training with individual LOS evaluated in advance and then promote COP movement in proximity to these stability limits. System validity was evaluated for the younger and mature cohorts.

Here, Section 2 describes the system configuration and processing components; Section 3 outlines the verification experiments; Section 4 presents the discussion; and Section 5 provides the conclusion and thoughts on future work.

## 2. Proposed System

### 2.1. Hardware

This study builds upon visual approaches, dynamic center of mass movement, and personalized training methodologies whose effectiveness has been demonstrated in previous research. The objective is to address the challenges of appropriate load determination, high cost and onerous system requirements.

**Figure 2:**
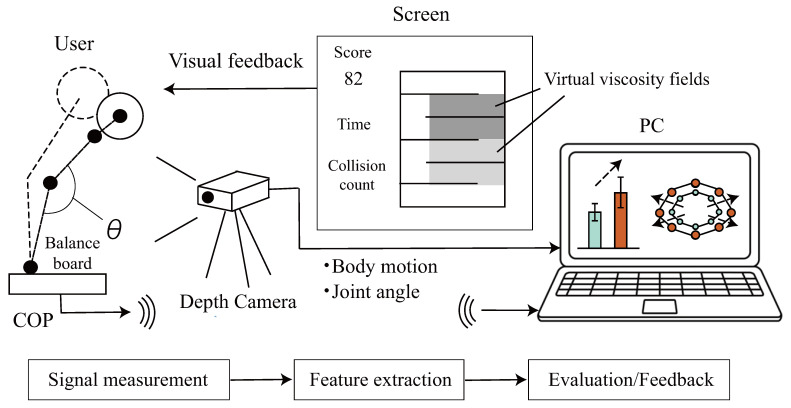
Proposed LOS evaluation/training system.

The proposed system is inexpensive and compact, allowing weight-shifting training based on individual stability limits using visual stimuli and applying individualized loads to enhance postural stability (Figure 2). The hardware configuration comprises a center of pressure (COP) measurement device (Wii Balance Board, Nintendo, Kyoto, Japan) [12,13], an Azure Kinect depth sensor (Microsoft, Redmond, WA, USA), a PC and a display screen.

Subjects standing on the COP device control virtual objects projected on the screen using their center of pressure. The system determines COP positions at sampling frequency $f_{s,\mathrm{COP}}$ and subject joint angles $\theta_{j=1,2,\ldots ,L}$ via the Azure Kinect at sampling frequency $f_{s,\mathrm{kinect}}$. The display information depends on whether limits of stability (LOS) evaluation or training protocols are being conducted.

### 2.2. Software: Evaluation Phase

**Figure 3:**
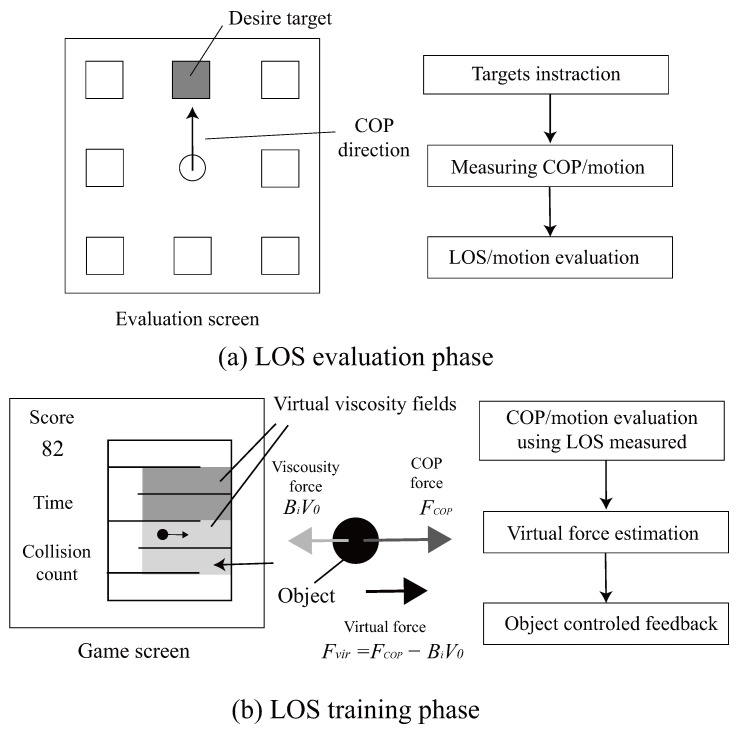
Software components for each phase.

The system software components for this phase are illustrated in Figure 3a, which shows how subjects modulate their COP to align a directional indicator with the designated target for measurement. Feature parameters for evaluating limits of stability, postural stability, and control strategies are initially extracted from the acquired COP and joint angle data. Stability limits for directional postural tilting are determined by quantifying the range within which subjects maintain postural equilibrium with a fixed base of support during maximal body lean in each specified direction. Following central COP position measurement, stability limit coordinates are acquired at stability boundaries across *D* directions within the horizontal plane, encompassing forward, backward, left, right, and diagonal orientations. In the acquisition protocol, the experimenter indicates target directions by changing a box-shaped object to red, subjects modulate their COP to align a directional indicator with the designated target. Measurement initiation occurs when the experimenter transitions the object to blue upon receiving the subject’s readiness signal. The measurement duration is established at 5 s. During data acquisition, the object transitions to green when COP oscillation amplitudes in both the frontal and sagittal planes remain within 5 cm thresholds. Upon the subject’s return of COP to the central position, the object displays gray coloration, signifying measurement completion for the specified direction. When oscillation amplitudes exceed the 5 cm threshold, the object appears as white, necessitating remeasurement. This protocol is repeated for all *D* directional measurements.

Stability limit area $A_{\mathrm{LOS}}$, COP sway rectangular area $S_{\mathrm{rect}}$, and postural stability index (IPS) values are computed from COP coordinate data ($x_{n}$, $y_{n}$) acquired during the evaluation phase. $x_{n}$ and $y_{n}$ correspond to the frontal and sagittal plane positions, respectively.

The stability limit area $A_{\mathrm{LOS}}$ is defined as the at encompassed by a *D*-sided polygon constructed by sequentially connecting the stability limit coordinates $\left(x_{i},y_{i}\right)$ for all *D* directional measurements (i=0,1,…,D−1) in clockwise sequence. This is computed as:

(1) $$A_{\mathrm{LOS}}=\frac{1}{2}\left|\sum_{i=0}^{D}\left(x_{i}y_{i+1}-x_{i+1}y_{i}\right)\right|$$

Here, when i=D, the calculation is performed with i=0.

IPS (Mochizuki et al. [14]) is calculated as:

(2) $$x_{\mathrm{amp},\;\mathrm{LOS}}=x_{\lim,\;\mathrm{right}}-x_{\lim,\;\mathrm{left}}$$

(3) $$y_{\mathrm{amp},\;\mathrm{LOS}}=y_{\lim,\;\mathrm{front}}-y_{\lim,\;\mathrm{back}}$$

(4) $$S_{\mathrm{rect},\;\mathrm{LOS}}=x_{\mathrm{amp},\;\mathrm{LOS}}y_{\mathrm{amp},\;\mathrm{LOS}}$$

(5) $$\mathrm{IPS}=\log\left(\frac{{\bar{S}}_{\mathrm{rect},\;\mathrm{dir}}+S_{\mathrm{rect},\;\mathrm{LOS}}}{{\bar{S}}_{\mathrm{rect},\;\mathrm{dir}}}\right)$$

Here, $S_{\mathrm{LOS}}$ represents the stability limit rectangular area, and ${\bar{S}}_{\mathrm{rect},\;\mathrm{dir}}$ represents the average COP sway rectangular area $S_{\mathrm{rect},\;\mathrm{dir}}$ for directions dir∈{center,front,left,back,right}.

### 2.3. Software: Training Phase

#### 2.3.1. In-Game Object Control

The game-based feedback system is detailed in Figure 3b, showing the virtual object, applied forces and virtual viscosity fields used during training. In the training phase, force is applied to a rod-shaped virtual object corresponding to the subject’s COP displacement, resulting in object movement. The session concludes when the object reaches the target goal located in the upper-left corner of the virtual environment. The applied force $F_{0}\left(x,y\right)=\left(F_{x},F_{y}\right)$ increases proportionally as the subject’s COP approaches individual stability limits, described as:

(6) $$F_{x}=\left\{\begin{array}{cc} \beta \left(x-x_{\lim,r}P_{2}\right)k_{x}\\ \;+\alpha x_{\lim,r}\left(P_{2}-P_{1}\right)k_{x} & \mathrm{if}\;x\geq x_{\lim,r}P_{2}\\ \alpha \left(x-x_{\lim,r}P_{1}\right)k_{x} & \mathrm{if}\;x_{\lim,r}P_{2}>x\geq x_{\lim,r}P_{1}\\ 0 & \mathrm{if}\;x_{\lim,r}P_{1}>x>x_{\lim,l}P_{1}\\ \alpha \left(x-x_{\lim,l}P_{1}\right)k_{x} & \mathrm{if}\;x_{\lim,l}P_{1}\geq x>x_{\lim,l}P_{2}\\ \beta \left(x-x_{\lim,l}P_{2}\right)k_{x}\\ \;+\alpha x_{\lim,l}\left(P_{2}-P_{1}\right)k_{x} & \mathrm{if}\;x_{\lim,l}P_{2}\geq x \end{array}\right.$$

$F_{y}$ follows the same definition with parameter substitutions x→y and r,l→f,b.

Here, (x,y) denote the current COP coordinates, $\left(y_{\lim,\;f},y_{\lim,\;b},x_{\lim,\;l},x_{\lim,\;r}\right)$ denote the stability limit positions in the front, back, left, and right directions, respectively, $P_{1},P_{2}$ are stability limit range coefficients, $k_{x},k_{y}$ are force scaling parameters, and α,β denote force gradient coefficients.

#### 2.3.2. Virtual Viscosity Field Control

The virtual environment incorporates a force field system in which viscous forces are applied in proportion to object velocity. The viscosity sensation is presented visually through the kinematic response of the virtual object, without relying on a physical force feedback device [15]. The game space is divided into *n* distinct viscous regions, each characterized by a viscosity coefficient $B_{i}$ (i=1,2,…,n).

The resulting viscous force $F_{vir}$ applied to the object is described as:

(7) $$F_{vir}=F_{cop}-B_{i}V_{0}$$

Here, $F_{cop}$ denotes the force generated by COP displacement, $V_{0}$ denotes instantaneous velocity, and $B_{i}$ denotes the viscosity coefficient for region *i*.

Object velocity *V* is updated using:

(8) $$\begin{array}{cc} V & =V_{0}+\frac{F_{vir}}{M}\Delta T \end{array}$$

(9) $$\begin{array}{cc} \; & =V_{0}+\frac{F_{cop}-B_{i}V_{0}}{M}\Delta T \end{array}$$

(10) $$\begin{array}{cc} \; & =\left(1-\frac{B_{i}}{M}\Delta T\right)V_{0}+\frac{\Delta T}{M}F_{cop} \end{array}$$

Here, *M* denotes the object mass and ΔT denotes the time interval. Modulation of the viscosity coefficient $B_{i}$ elicits varying degrees of COP displacement in subjects, ranging from subtle to more substantial movements.

#### 2.3.3. Evaluation/Feedback

Subjects were instructed to navigate the displayed route as quickly as possible while avoiding wall collisions. Performance evaluation was conducted based on completion time and route optimization, with real-time visual feedback provided during gameplay. COP movement analysis involved computation of central axis crossings as a sway frequency indicator (${cross}_{x}$, ${cross}_{y}$), and temporal distribution across three LOS-boundary-distance regions (central: <25%; intermediate: 25–75%; peripheral: ≧75%).

## 3. Experiment

### 3.1. Analysis

To eliminate any influence from outliers that might obscure overall index trends (such as those caused by significant balance disturbances), removal was performed as outlined here. The interquartile range (IQR) was calculated using the following equation with data values for each index arranged in descending order:

(11) IQR=Q3−Q1

Here, Q3 and Q1 represent the third and first quartiles, respectively.

The value of *n* after outlier removal was calculated as:

$$n=\left|\left\{\;x\;|\;Q1-1.5\times IQR\;\leq \;x\;\leq \;Q3+1.5\times IQR\right\}\right|.$$

Here, *x* denotes the index value.

Statistical testing for each index is described below. For the stability limit area $A_{\mathrm{LOS}}$, COP sway rectangular area $S_{\mathrm{rect}}$ and IPS, paired *t*-tests were used for normally distributed data, while Wilcoxon signed-rank testing was applied to non-normal data. For the crossing counts ${cross}_{x}$ and ${cross}_{y}$ and the COP position ratio $R_{\mathrm{cop},\;x}$, independent *t*-testing was applied for normally distributed data, while Mann–Whitney U testing was applied to non-normal data. Pearson’s correlation coefficient was used for normally distributed continuous data, Spearman’s rank correlation coefficient for non-normal continuous data, and Kendall’s rank correlation coefficient for discrete data. Detailed statistical results including effect sizes and 95% confidence intervals are provided in Supplementary Materials (Tables S1–S8).

### 3.2. Initial Effect Verification Experiment

#### 3.2.1. Experimental Conditions

**Figure 4:**
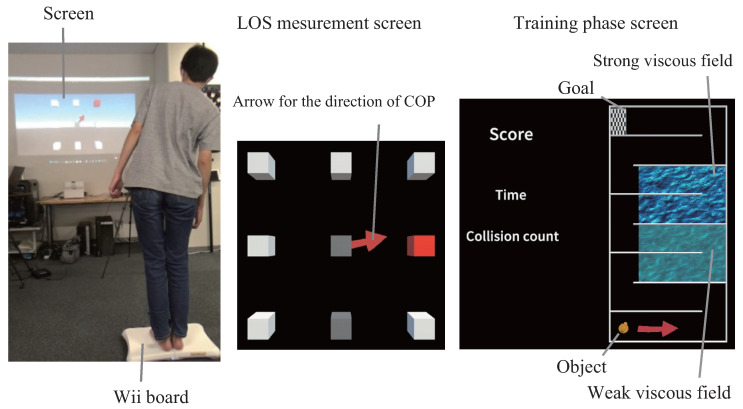
Screen set-up.

**Figure 5:**
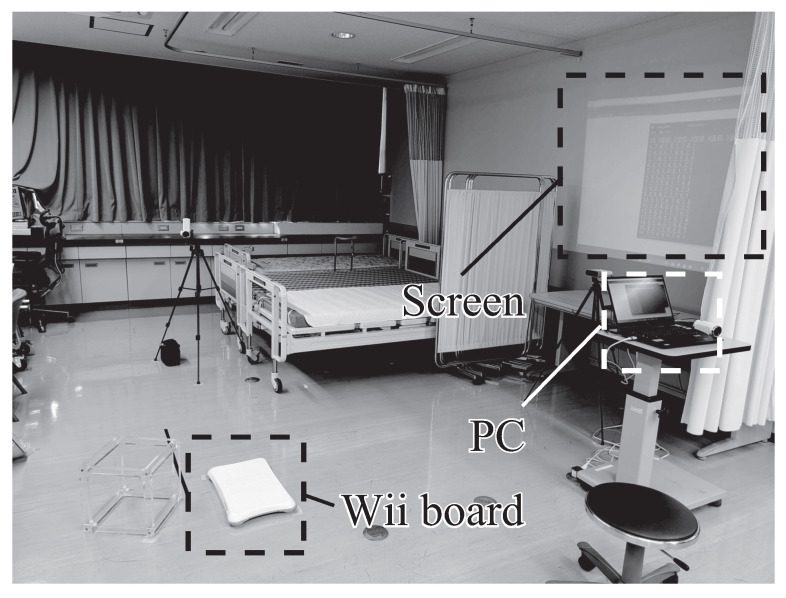
Experiment set-up.

The initial effects of postural stability enhancement from the proposed system were assessed in 32 healthy young adults (13 males, 19 females; mean age 21.4 ± 2.4 years) and 19 healthy mature adults (10 males, 9 females; mean age 73.1 ± 9.2 years). The system comprised a Wii Balance Board ($f_{s,\mathrm{COP}}$ = 100 Hz), an Azure Kinect depth sensor ($f_{s,\mathrm{kinect}}$ = 30 Hz), and a display device (projector/TV). The measurement conditions and virtual space displayed during each phase are shown in Figure 4, and the experimental setup is shown in Figure 5. The experimental parameters for each phase were set as: stability limit range coefficients: $P_{1}$ = 0.25, $P_{2}$ = 0.75; force scaling coefficients: $k_{x}$ = 0.025, $k_{y}$ = 0.00375; force change rates: α = 0.25, β = 1.0; viscosity coefficients: $c_{0}$ = 0.3, $c_{1}$ = 0.1, $c_{2}$ = 2/3; number of measurement directions: *D* = 8.

The experimental design comprised the sequential phases of evaluation, training and re-evaluation. Each training session required approximately 1–2 min for younger participants and 1–3 min for mature participants to complete, with completion time varying by individual. Evaluation phases were conducted immediately before and after the training session. Participants were healthy individuals with no known neurological, orthopedic, or visual impairments that could affect balance or performance. The younger cohort completed additional physical fitness evaluations to investigate whether individual physical abilities contribute to stability and training performance. These assessments encompassed toe-grip strength, sit-and-reach flexibility, ankle range of motion, functional reach test (FRT), two-step test, five m balance beam walk, and single-leg standing test [16], and their relationships with the evaluation indices of the proposed system were examined. Toe-grip strength was measured twice for each foot, while the two-step test and five m balance beam walk were performed in two trials each, with the best performance selected for analysis.

#### 3.2.2. Results

**Figure 6:**
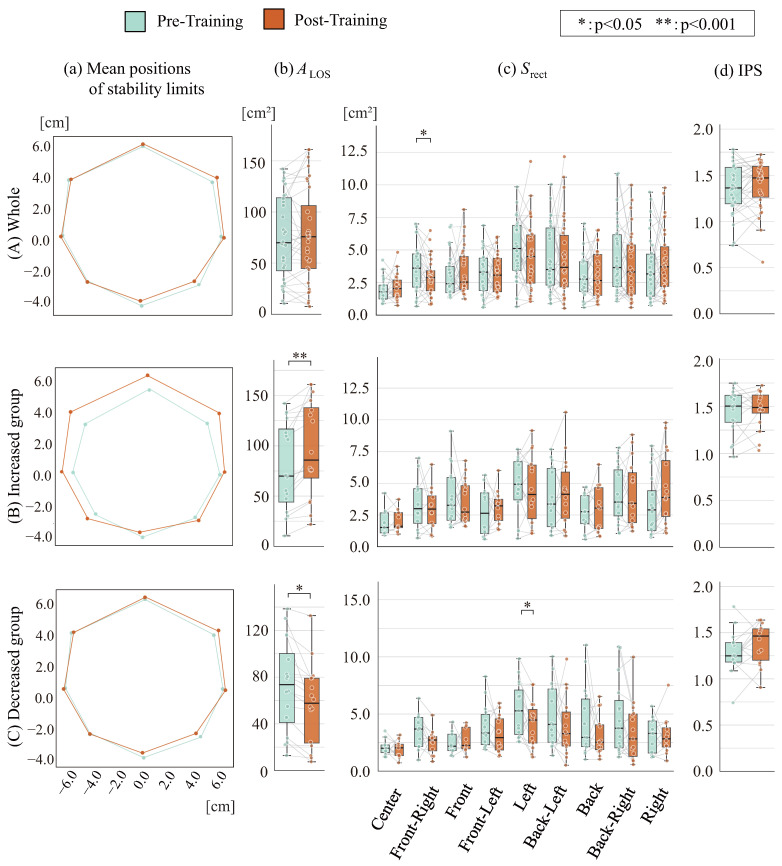
Initial effects among younger cohort.

**Figure 7:**
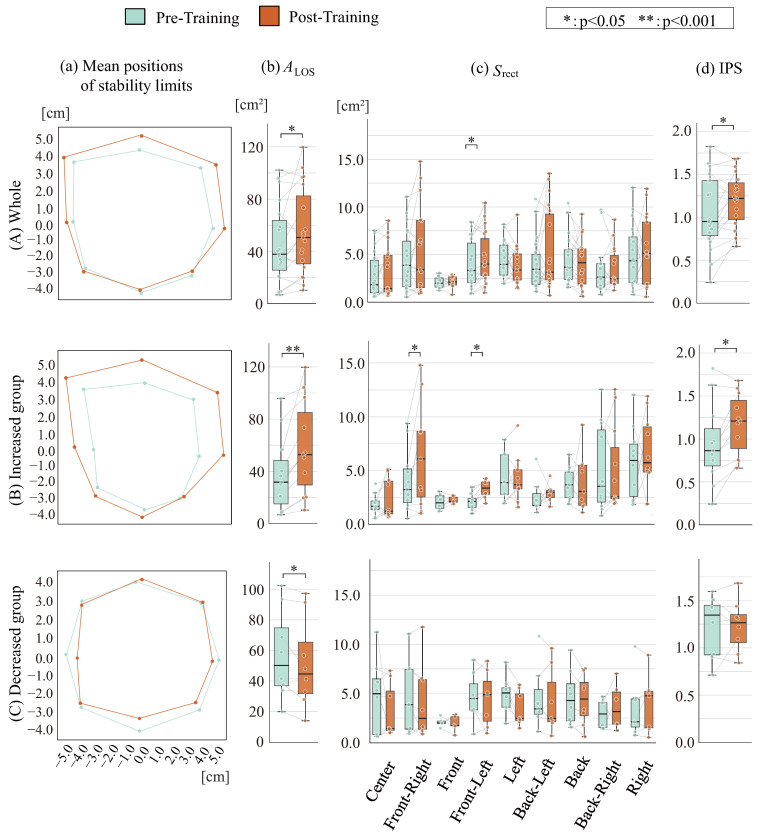
Initial effects among mature cohort.

Figure 6A(a) presents the stability limit positions for the younger cohort, while Figure 6A(b),A(c),A(d) display the stability limit area $A_{\mathrm{LOS}}$, the COP sway rectangular area $S_{\mathrm{rect}}$, and IPS values, respectively. Similarly, Figure 7A(a) shows the stability limit positions for the mature cohort, and Figure 7A(b),A(c),A(d) present the corresponding stability limit area $A_{\mathrm{LOS}}$, COP sway rectangular area $S_{\mathrm{rect}}$, and IPS values, respectively. Figure 6A(a),A(b) show no significant post-training trend in stability limit position or area for the younger cohort. However, Figure 6A(c) demonstrates a decrease in the COP sway rectangular area at the right-forward position. Figure 6A(d) shows a slight increase in IPS values, although no statistical significance is observed. For the mature cohort, Figure 7A(a) demonstrates post-training expansion of stability limit positions in the left-right direction and the three forward directions. Figure 7A(b),A(c) show a significant increase in stability limit area, while no trend was observed in the COP sway rectangular area. Consequently, Figure 7A(d) demonstrates a significant increase in IPS.

Post-training changes in IPS were observed across both age cohorts. Analysis revealed two distinct improvement strategies differentiated by stability limit area modifications. Subjects were subsequently stratified into groups exhibiting increased stability limit area (younger: *n* = 16, mature: *n* = 11) versus decreased area (younger: *n* = 16, mature: *n* = 8). Corresponding results are seen in Figure 6B,C and Figure 7B,C.

Figure 6B(a) and Figure 7B(a) demonstrate that in the group with increased stability limit area, post-training stability limit positions were extended in the left-right direction and the three forward directions. Figure 6B(b) and Figure 7B(b) show a significant increase in stability limit area. Figure 6B(c) and Figure 7B(c) show that while no trend was observed in the COP sway rectangular area for the younger group, a significant increase was noted in the left-forward and right-forward positions for the mature group. Figure 6B(d) and Figure 7B(d) indicate an increasing trend in IPS values for the younger group and a significant increase for the mature group.

Figure 6C(a) and Figure 7C(a) demonstrate post-training reduction in stability limit positions. Figure 6C(b) and Figure 7C(b) show a significant decrease in stability limit area. Figure 6C(c) and Figure 7C(c) show that for the younger group, an overall decreasing trend was observed in the COP sway rectangular area, with significant decreases in the right-forward and left directions. For the mature group, no consistent trend was observed. Figure 6C(d) and Figure 7C(d) indicate no significant change in IPS values.

**Figure 8:**
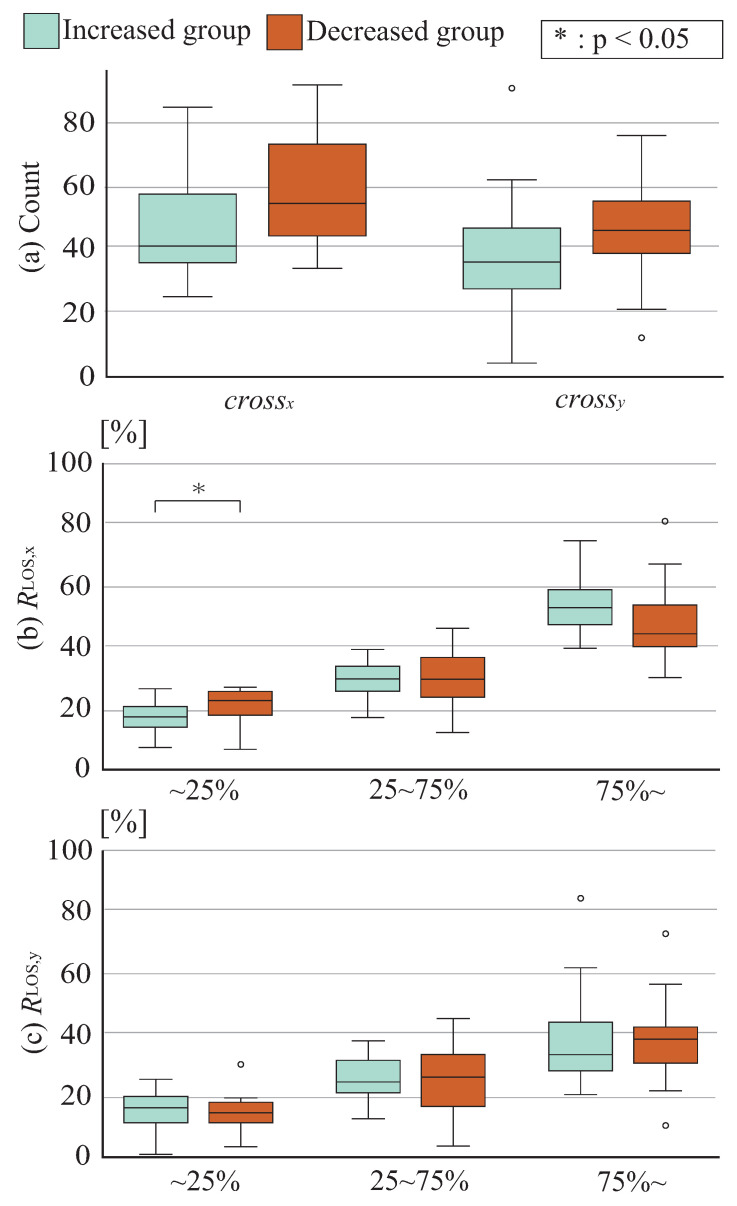
Training phase indicators among younger cohort.

**Figure 9:**
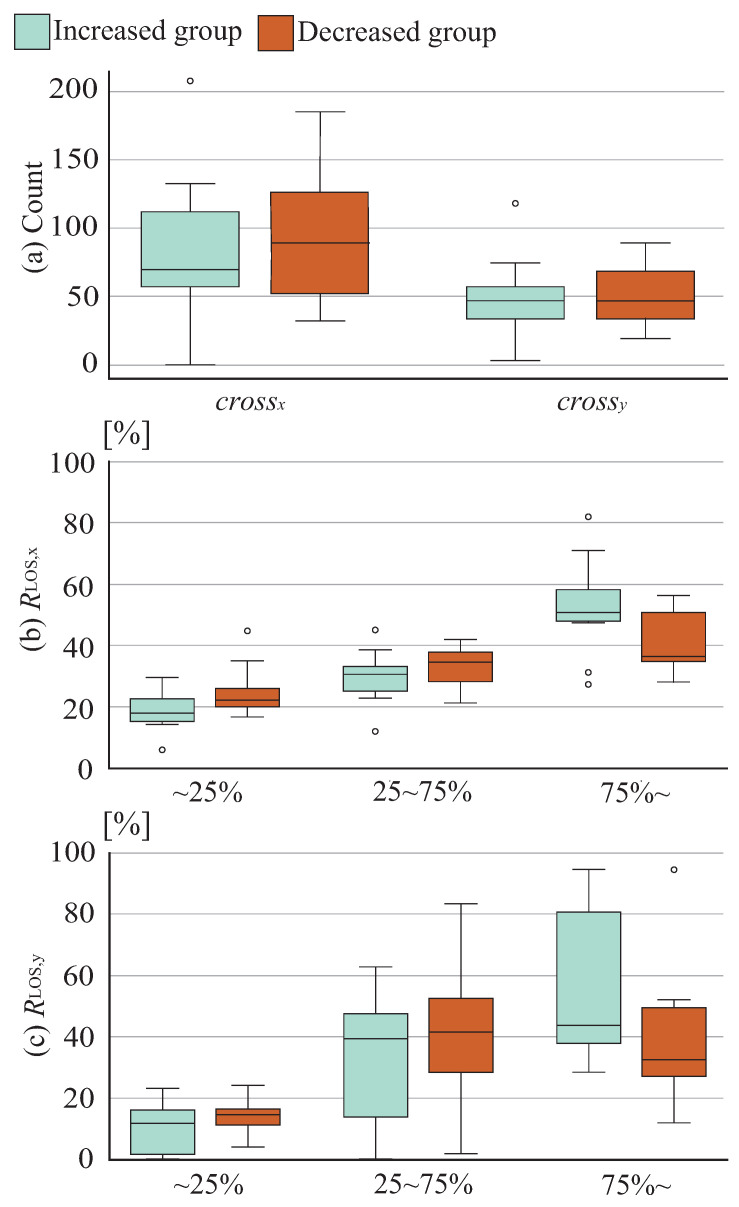
Training phase indicators among mature cohort.

Figure 8 and Figure 9 show numbers of crossings and the temporal ratio of COP positions in the x and y directions during the training phase. (a) shows numbers of crossings, while (b) and (c) show temporal ratios of COP positions in the x and y directions, respectively. The numbers of crossings ${cross}_{x}$ and ${cross}_{y}$ in COP movement patterns tended to be smaller in the increase group than in the reduction group. For the younger group, the COP position ratio in the x direction $R_{\mathrm{cop},x}$ showed a larger tendency in the increase group at 75% or greater and significantly smaller values at 25% or less. No marked trend difference was observed between groups for the ratio in the y, and no significant difference was observed in either the x or y directions for the mature group, while the increase group showed a tendency toward larger values at 75% or greater and smaller values at 25% or less. These results demonstrate that the stability limit area increase group exhibited relatively more COP movement near stability limit positions and fewer returns to the central position.

**Figure 10:**
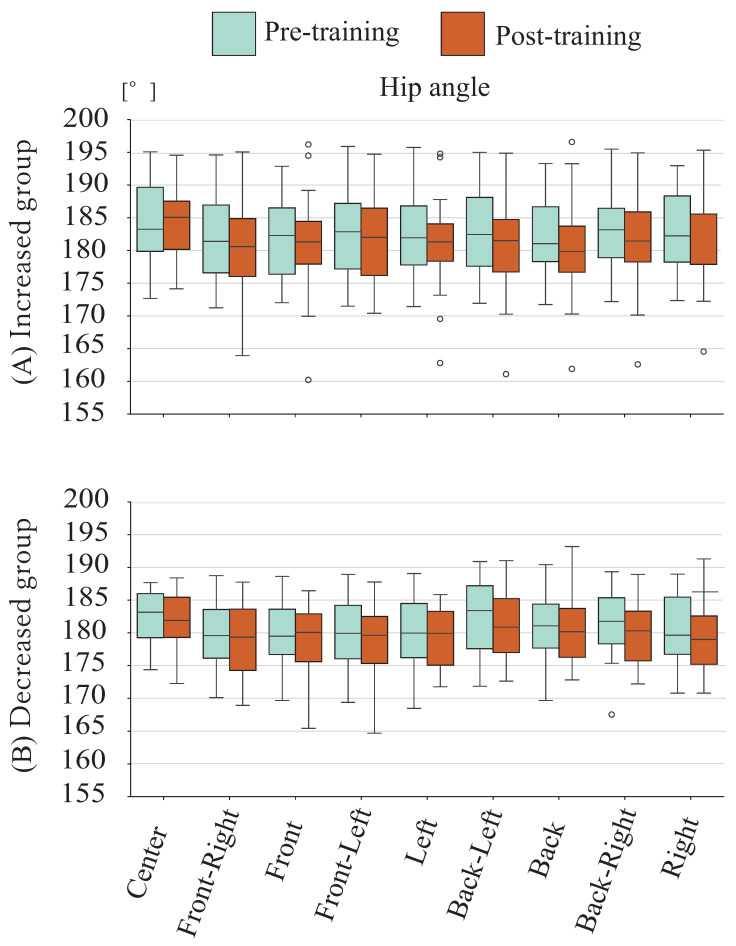
Hip angle during evaluation phase among younger cohort.

Figure 10 shows mean hip joint angles in the sagittal plane during stability limit evaluation for the groups with increased and decreased stability limit area. In both groups, a decreasing trend in angles was observed in each direction after training. The increase group also showed a larger tendency toward greater angle reduction in the forward direction than the decrease group.

Table 1 and Table 2 show correlations between pre- and post-training IPS, IPS increase and physical fitness tests. In the increased stability limit area group, a significant strong negative correlation was observed between pre-training IPS and time in the balance beam test, as well as a moderate positive correlation with left and right toe-grip strength. Post-training IPS showed a significant moderate negative correlation with time in the balance beam test, while the IPS increase rate exhibited a moderate negative correlation with right toe-grip strength. In the decreased stability limit area group, pre-training IPS showed a significant moderate negative correlation with sit-and-reach flexibility, while no correlation was observed with post-training IPS. The IPS increase rate showed a significant moderate positive correlation with the range of motion (extension) of the right ankle joint.

### 3.3. Evaluation of Ongoing Effects Among Younger Cohort

#### 3.3.1. Experimental Conditions

As a pilot study, this experiment was designed to exploratorily investigate the tendency of effects on postural stability through ongoing training, and the influence of fatigue accumulation throughout that process. The sustained effects of stability improvement through training with the proposed system were evaluated in five healthy young males (mean age 22.8 ± 1.3 years) using a Wii Balance Board and a television. Participants performed one training session per day for four consecutive days, with evaluation phases conducted immediately before and after each training session. The system parameters for the training phase were: stability range coefficients $P_{1}$ = 0.25 and $P_{2}$ = 0.75, force scaling coefficients $k_{x}$ = 0.025 and $k_{y}$ = 0.00375, force variation rates α = 0.25 and β = 1.0, viscosity coefficients $c_{0}$ = 0.3, $c_{1}$ = 0.1 and $c_{2}$ = 2/3, and number of measurement directions: *D* = 8. To assess the potential effects of training and daily fatigue accumulation on stability limit size and COP sway, Visual Analogue Scale (VAS) data were collected immediately after the first evaluation phase, training phase, and second evaluation phase. VAS is widely used for quantifying subjective sensations and emotions, such as fatigue or pain, by recording their intensity on a linear scale [17]. Subjects marked their perceived sensation on the scale from “Not tired at all (0)” to “Extremely tired (100).” To evaluate whether improvements in static standing balance were observed before and after training, balance ability was assessed using the StA^2^BLE approach developed by Shima et al. [18], which evaluates subjects’ standing balance based on age models by inducing COP sway. The equipment used both measures muscle strength and enables evaluation of sensory systems, which are otherwise challenging to assess.

#### 3.3.2. Results

**Figure 11:**
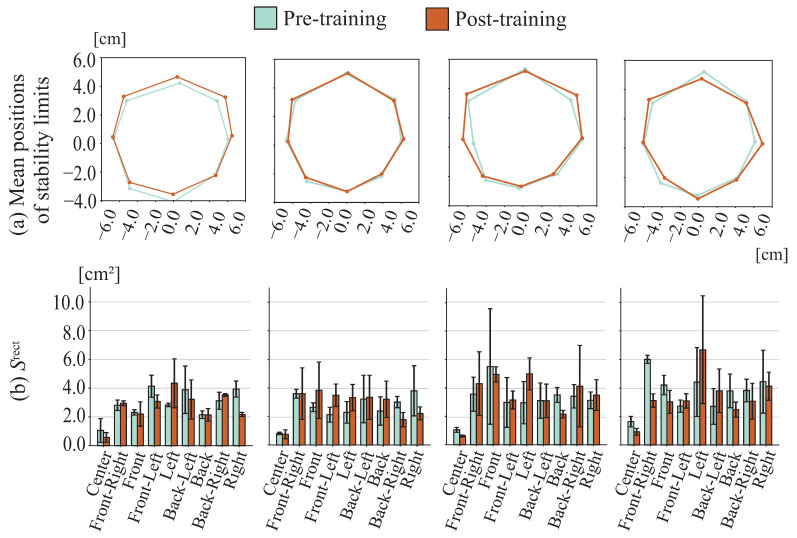
Ongoing effects for COP position among younger cohort.

**Figure 12:**
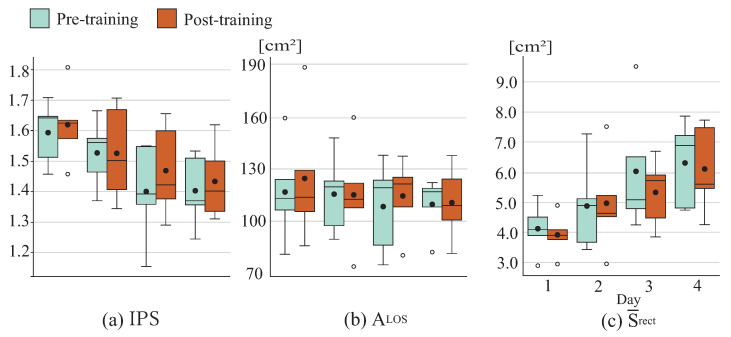
Ongoing effects among younger cohort.

Figure 11 shows stability limit positions and the COP sway rectangular area $S_{\mathrm{rect}}$ for each direction. Figure 12 displays the stability limit area $A_{\mathrm{LOS}}$, the COP sway rectangular area $S_{\mathrm{rect}}$, and IPS, including changes before and after training and across days. No clear trends were observed in stability limit positions either immediately after or across days of training. A tendency toward initial improvement was observed in the stability limit area $A_{\mathrm{LOS}}$, although sustained improvement tendencies across days were limited. A tendency toward initial improvement was also observed in the mean COP sway rectangular area $S_{\mathrm{rect}}$, although this tendency appeared to diminish across days. IPS, thus, showed a tendency to increase immediately after training, although this improvement tendency was not maintained across days.

**Figure 13:**
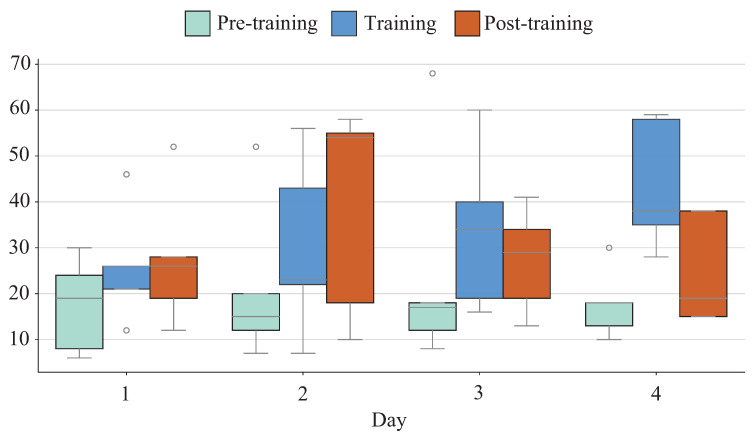
VAS results in ongoing training among younger cohort.

VAS results are shown in Figure 13. Fatigue levels immediately after the first evaluation phase showed little change, while those the training phase increased on a daily basis. Levels immediately after the second evaluation phase peaked on the second day, forming a bell-shaped trend.

**Figure 14:**
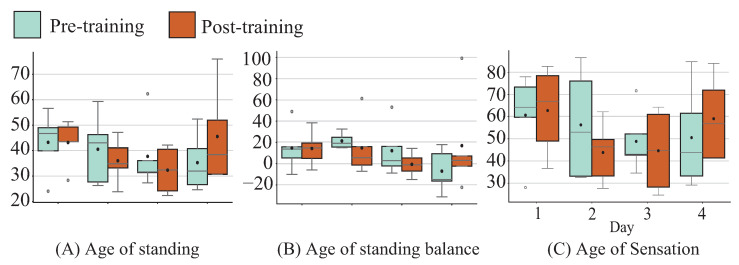
Continued effects for balance ability (StA^2^BLE) among younger cohort.

Figure 14 shows StA^2^BLE evaluation. For standing age, balance age and sensory age, there was a tendency for initial and sustained decreases except after the final day of training.

## 4. Discussion

### 4.1. Observed Response Patterns

The experimental results suggest that changes in postural stability were associated with two distinct response patterns. It should be noted that these two patterns represent observed response profiles identified through post hoc classification based on outcome changes, rather than predefined training strategies. This approach is exploratory in nature and should be interpreted with caution.

#### 4.1.1. LOS Expansion Pattern

The LOS expansion strategy group demonstrated stability limit area increases consistent with Vassilis et al.’s weight-shifting training findings [9]. Training-induced muscle activity enhancement likely strengthened ankle joint proprioceptive input, thereby improving plantar and dorsiflexor eccentric contraction capacity. Increased activity in the triceps surae muscles, such as the soleus and gastrocnemius, has been reported to cause the COP to shift forward [19]. Since the proposed system facilitates forward COP movement, enhanced triceps surae activation likely contributed to forward stability limit expansion. Analogously, left-right movement phases may have activated trunk lateral flexors, promoting bilateral stability limit extension.

#### 4.1.2. COP Sway Reduction Pattern

The sway reduction strategy likely represents a compensatory mechanism whereby subjects minimized postural oscillations while accepting reduced stability boundaries. Wang et al. reported that mechanically enhancing plantar sensation improves postural stability in elderly populations, indicating that plantar skin input contributes to reduced postural sway [20]. Since the proposed system promotes multidirectional COP displacement, enhanced plantar stimulation may have improved sensory acuity, consequently reducing COP variability.

### 4.2. Postural Control Strategies and Age-Related Differences

Postural control strategies include approaches in which body sway is mitigated using the ankle joints or hip joints [21]. It has previously been reported that healthy young adults use the ankle strategy for COP movements within the range close to the center of LOS, but switch to the hip strategy for COP movements closer to its outer limits [22]. For a mixed approach combining both strategies [23], it has been reported that LOS are extremely narrow in Parkinson’s disease patients with unstable posture, and mixed synergy effects are observed within the LOS range [22]. This suggests that the mechanism of stability change from a postural strategy perspective may depending on whether the subject’s stability limit area increases or decreases.

In older adults, the stability limit correlates with fear of falling, and is constrained by subject perception [24]. The mature cohort showed more pronounced group differences than the younger one at the stability limit periphery (≥75%) and in the central region (<25%), with significant differences also observed in the intermediate range (25–75%). This suggests that age-related decline in standing function and fear of falling may have increased the proportion of COP movements within the intermediate range, where fall risk is perceived as lower.

### 4.3. Individual Variability Factors

Physical fitness correlation analysis for the younger cohort demonstrated strategy-specific selection patterns based on individual capabilities. Subjects exhibiting superior lower-limb strength (toe grip, balance beam performance) preferentially utilized LOS expansion strategies, while those with enhanced flexibility (sit-and-reach, ankle range of motion) favored sway reduction approaches. These phenotypic variations in physical characteristics appear to underlie individual strategic preferences.

The mature cohort exhibited more pronounced improvement than the younger one. This likely reflects diminished baseline stability, creating enhanced adaptive capacity and making training effects more apparent.

### 4.4. Sustained Training Effects and Fatigue

It should be noted that the sustained-effect experiment was conducted as a pilot study, and the following findings should, therefore, be interpreted as exploratory in nature. In the sustained-effect experiment, a tendency toward initial improvement was observed; however, this improvement tendency appeared not to be maintained across days. VAS-based fatigue evaluation suggests that physical condition on the day may have influenced training effects, as daily fatigue levels during training increased progressively throughout the experiment. Muscular fatigue has been shown to impair postural stability [25,26,27]. Conversely, static standing balance assessment using StA^2^BLE showed a tendency toward sustained improvement. This may be attributed to enhanced sensory perception from plantar stimulation, with effects more apparent in static standing due to reduced sensitivity to fatigue than in maintenance of positions near stability limits. As a lesson learned from these exploratory findings, the design of training protocols incorporating adequate rest periods is suggested as an important direction for future work.

### 4.5. Limitations

Several important limitations of this study should be acknowledged. The absence of a control condition means that comparisons with alternative intervention approaches or repeated measurement effects have not been performed; quantitative evaluation of the effects specific to the proposed system, therefore, remains a direction for future work. It should be noted, however, that the absence of a control condition does not negate the effectiveness of the proposed system, given that the observed improvements occurred in the context of training with the system. Second, the sustained-effect experiment was conducted as a pilot study with only five participants, which is insufficient to ensure adequate statistical power; the findings should, therefore, be interpreted as preliminary and exploratory. Third, subgroup analyses in the initial experiment stratifying participants into LOS increase and decrease groups were conducted as a post hoc exploratory analysis to understand participants who showed different response patterns. As the classification was based on observed outcomes rather than predefined criteria, these findings are exploratory in nature and require validation in future studies with prospectively defined subgroups. Furthermore, these analyses substantially reduced effective sample sizes, and comparisons within the mature cohort in particular should be interpreted with caution. Fourth, the study involves multiple outcome measures (LOS area, COP sway, IPS, etc.) and subgroup analyses without adjustment for multiple comparisons, which increases the risk of Type I error. The findings should be interpreted accordingly, particularly for subgroup comparisons and exploratory analyses. Finally, the physiological mechanisms discussed such as increased triceps surae activation, enhanced proprioceptive input, plantar sensory facilitation, and shifts between ankle and hip strategies were not directly measured via EMG or sensory testing, and these explanations should, therefore, be regarded as speculative. It should also be noted that the Wii Balance Board used in this study has been discontinued by the manufacturer. Although the software architecture of the proposed system is designed to interface with any device capable of transmitting COP data in real time via Bluetooth and does not depend on hardware specifications unique to the Wii Balance Board, transitioning to an alternative device would require device-specific calibration and accuracy validation. In light of these limitations, the findings regarding sustained effects and subgroup analyses should be interpreted as preliminary and exploratory, and future work should include an increased number of subjects, the incorporation of control conditions, and mechanistic investigations using electromyographic evaluation.

## 5. Conclusions

The authors proposed a personalized training system based on individual limits of stability (LOS) to help prevent falls among the elderly. The system utilizes a center of pressure (COP) evaluation device and game-based visual feedback, determining LOS in eight directions and subsequently promoting COP movement in proximity to stability limits to achieve individualized training loads.

Verification experiments showed post-training changes in the postural stability index (IPS) for both the younger and mature cohorts. Particularly significant was the observation of two distinct improvement strategies based on changes in stability limit area: a stability limit area expansion group (extension of stability limits) and a sway reduction group (refined control of COP oscillation). Correlation analysis with physical fitness tests revealed that subjects with superior lower-limb strength tended to adopt the LOS expansion strategy, while those with greater flexibility favored the sway reduction strategy. The mature cohort exhibited more pronounced improvement than the younger cohort, suggesting that lower baseline stability provided greater potential for enhancement.

The sustained effect experiment revealed initial improvement effects, while demonstrating that daily fatigue accumulation may limit sustained improvements. However, static standing balance showed sustained improvement trends, suggesting effects from enhanced plantar sensory perception. These results highlight the importance of training protocols that incorporate adequate rest periods.

These preliminary findings suggest that personalized training based on individual LOS is associated with response patterns tailored to individual physical characteristics. Future work will include increasing the number of subjects, validating long-term training protocols with optimal rest intervals, and conducting detailed analysis of improvement mechanisms using electromyographic evaluation. In future work, combining the proposed system with hardware-based interventions, such as AI-driven personalized orthotic insole design [28], may offer a more comprehensive approach to addressing postural instability. 

## Figures and Tables

**Table 1:** Correlation between IPS and physical tests for increase group among younger cohort.

Item	IPS Increase Rate	IPS (Post-Training)	IPS (Pre-Training)
FRT	−0.186	−0.193	0.072
Right Foot Grip Strength	−0.443	0.152	0.421
Left Foot Grip Strength	−0.355	0.284	0.468
Right Single-leg Standing test	0.024	−0.306	−0.330
Left Single-leg Standing test	-	-	-
2-step	−0.396	0.110	0.426
Balance Beam Walk	0.291	−0.622 *	−0.735 **
Right Ankle: Flexion	−0.089	0.129	0.168
Left Ankle: Flexion	−0.028	0.009	0.009
Right Ankle: Extension	0.030	0.130	0.070
Left Ankle: Extension	−0.057	0.076	0.057
Seated Forward Flexion	0.015	−0.131	0.034

* *p* < 0.05; ** *p* < 0.01.

**Table 2:** Correlation between IPS and physical tests for decrease group among younger cohort.

Item	IPS Increase Rate	IPS (Post-Training)	IPS (Pre-Training)
FRT	−0.337	−0.274	0.143
Right Foot Grip Strength	0.253	0.172	−0.009
Left Foot Grip Strength	0.250	0.090	−0.003
Right Single-leg Standing test	0.254	0.274	0.071
Left Single-leg Standing test	0.254	0.254	0.017
2-step	−0.227	−0.021	−0.057
Balance Beam Walk	−0.082	0.138	0.356
Right Ankle: Flexion	0.366	0.089	−0.109
Left Ankle: Flexion	0.383	0.055	−0.183
Right Ankle: Extension	0.532 **	0.300	−0.048
Left Ankle: Extension	0.242	−0.093	−0.242
Seated Forward Flexion	−0.086	−0.383	−0.498 *

* *p* < 0.05; ** *p* < 0.01.

## Data Availability

The data presented in this study are available on request from the corresponding authors.

## Supplementary Materials

The following supporting information can be downloaded at: https://www.mdpi.com/article/10.3390/s26061985/s1, Table S1: Detailed statistical results for the younger cohort (whole group); Table S2: Detailed statistical results for the younger cohort (increased group); Table S3: Detailed statistical results for the younger cohort (decreased group); Table S4: Detailed statistical results for the mature cohort (whole group); Table S5: Detailed statistical results for the mature cohort (increased group); Table S6: Detailed statistical results for the mature cohort (decreased group); Table S7: Detailed statistical analysis of training phase indicators for the younger cohort; Table S8: Detailed statistical analysis of training phase indicators for the mature cohort.
