# Supplementary material for: A Training System for Human Standing Stability Using Virtual Viscosity Fields
Source: Sensors (Basel). 2026 Mar 22;26(6):1985. doi: 10.3390/s26061985 (PMC13030371; doi:10.3390/s26061985)
Supplement: Supplementary file 1 [file sensors-26-01985-s001.zip › sensors-4121320-supplementary.pdf]

Table S1. Detailed statistical results for the younger cohort (whole group) in the immediate effect verification experiment, including p-values and effect sizes

Index	Direction	Timing	Mean (SD)	Difference Mean (SD)	95% CI	p	Effect Size Type	Effect Size
IPS	–	Pre	1.346 (0.267)	0.025 (0.243)	[-0.20, 0.48]	0.432	Rank-biserial correlation	0.16
		Post	1.370 (0.313)					
A_{LOS}	–	Pre	75.672 (41.466)	1.042 (23.730)	[-0.32, 0.40]	0.806	Cohen's d_z	0.04
		Post	76.714 (46.069)					
S_{rect}	Center	Pre	3.216 (2.646)	-0.991 (2.534)	[-0.65, 0.02]	0.100	Rank-biserial correlation	-0.37
		Post	2.225 (0.936)					
	Front-Right	Pre	4.041 (2.420)	-0.946 (1.882)	[-0.92, -0.08]	0.015	Cohen's d_z	-0.50
		Post	3.095 (1.648)					
	Front	Pre	3.724 (2.519)	-0.368 (3.049)	[-0.41, 0.33]	0.831	Rank-biserial correlation	-0.05
		Post	3.356 (1.827)					
	Front-Left	Pre	3.629 (2.337)	-0.473 (2.471)	[-0.59, 0.21]	0.329	Cohen's d_z	-0.19
		Post	3.156 (1.443)					
	Left	Pre	5.122 (2.335)	-0.549 (2.303)	[-0.62, 0.14]	0.202	Cohen's d_z	-0.24
		Post	4.573 (2.706)					
	Back-Left	Pre	4.324 (2.598)	0.039 (3.452)	[-0.42, 0.29]	0.730	Rank-biserial correlation	-0.08
		Post	4.363 (3.010)					
	Back	Pre	3.158 (1.989)	-0.045 (2.129)	[-0.35, 0.42]	0.861	Rank-biserial correlation	0.04
		Post	3.112 (1.798)					
	Back-Right	Pre	4.696 (3.126)	-0.865 (2.819)	[-0.57, 0.09]	0.195	Rank-biserial correlation	-0.27
		Post	3.830 (2.537)					
	Right	Pre	3.817 (2.741)	0.176 (3.038)	[-0.26, 0.44]	0.622	Rank-biserial correlation	0.10
		Post	3.993 (2.459)					

Table S2. Detailed statistical results for the younger cohort (increased group) in the immediate effect verification experiment, including p-values and effect sizes

Index	Direction	Timing	Mean (SD)	Difference Mean (SD)	95% CI	p	Effect Size Type	Effect Size
IPS	–	Pre	1.443 (0.239)	0.028 (0.183)	[-0.39, 0.69]	0.556	Cohen's d_z	0.15
		Post	1.471 (0.205)					
A_{LOS}	–	Pre	78.784 (44.105)	18.151 (15.411)	[0.47, 1.88]	< .001	Cohen's d_z	1.18
		Post	96.935 (47.858)					
S_{rect}	Center	Pre	3.529 (2.778)	-1.348 (2.484)	[-0.78, 0.19]	0.216	Rank-biserial correlation	-0.41
		Post	2.181 (0.826)					
	Front-Right	Pre	3.825 (2.500)	-0.758 (2.252)	[-0.96, 0.29]	0.248	Cohen's d_z	-0.34
		Post	3.067 (1.578)					
	Front	Pre	3.886 (2.259)	-0.402 (2.478)	[-0.55, 0.44]	0.821	Rank-biserial correlation	-0.07
		Post	3.484 (1.730)					
	Front-Left	Pre	3.323 (2.755)	-0.251 (3.084)	[-0.62, 0.48]	0.787	Rank-biserial correlation	-0.10
		Post	3.071 (1.302)					
	Left	Pre	4.559 (2.292)	0.550 (3.246)	[-0.39, 0.73]	0.522	Cohen's d_z	0.17
		Post	5.110 (3.427)					
	Back-Left	Pre	3.822 (2.477)	0.479 (2.460)	[-0.39, 0.78]	0.479	Cohen's d_z	0.19
		Post	4.301 (2.786)					
	Back	Pre	2.582 (1.382)	0.336 (1.505)	[-0.39, 0.84]	0.436	Cohen's d_z	0.22
		Post	2.918 (1.863)					
	Back-Right	Pre	4.167 (2.293)	0.079 (2.189)	[-0.52, 0.59]	0.890	Cohen's d_z	0.04
		Post	4.246 (2.586)					
	Right	Pre	3.249 (2.394)	1.286 (2.914)	[-0.01, 0.79]	0.093	Rank-biserial correlation	0.49
		Post	4.535 (2.879)					

Table S3. Detailed statistical results for the younger cohort (decreased group) in the immediate effect verification experiment, including p-values and effect sizes

Index	Direction	Timing	Mean (SD)	Difference Mean (SD)	95% CI	p	Effect Size Type	Effect Size
IPS	–	Pre	1.248 (0.266)	0.021 (0.298)	[-0.36, 0.61]	0.597	Rank-biserial correlation	0.16
		Post	1.270 (0.373)					
A_{LOS}	–	Pre	72.560 (39.844)	-16.068 (17.371)	[-1.57, -0.28]	0.002	Cohen's d_z	-0.92
		Post	56.493 (34.980)					
S_{rect}	Center	Pre	1.973 (0.754)	0.183 (1.537)	[-0.52, 0.76]	0.688	Cohen's d_z	0.12
		Post	2.156 (1.108)					
	Front-Right	Pre	3.527 (1.570)	-0.966 (1.542)	[-1.33, 0.07]	0.053	Cohen's d_z	-0.63
		Post	2.561 (1.104)					
	Front	Pre	4.083 (3.478)	-0.881 (4.159)	[-0.63, 0.46]	0.735	Rank-biserial correlation	-0.12
		Post	3.202 (1.929)					
	Front-Left	Pre	3.914 (1.934)	-0.679 (1.828)	[-0.97, 0.23]	0.188	Cohen's d_z	-0.37
		Post	3.235 (1.609)					
	Left	Pre	5.402 (2.410)	-1.288 (2.207)	[-1.19, 0.02]	0.040	Cohen's d_z	-0.58
		Post	4.115 (2.052)					
	Back-Left	Pre	4.871 (2.759)	-0.971 (3.469)	[-0.85, 0.29]	0.297	Cohen's d_z	-0.28
		Post	3.900 (2.643)					
	Back	Pre	5.016 (3.990)	-1.548 (3.786)	[-0.75, 0.16]	0.208	Rank-biserial correlation	-0.38
		Post	3.468 (2.145)					
	Back-Right	Pre	5.191 (3.754)	-1.751 (3.114)	[-0.76, 0.09]	0.144	Rank-biserial correlation	-0.43
		Post	3.440 (2.509)					
	Right	Pre	4.424 (3.033)	-1.009 (2.786)	[-0.71, 0.23]	0.303	Rank-biserial correlation	-0.32
		Post	3.415 (1.839)					

Table S4. Detailed statistical results for the mature cohort (whole group) in the immediate effect verification experiment, including p-values and effect sizes

Index	Direction	Timing	Mean (SD)	Difference Mean (SD)	95% CI	p	Effect Size Type	Effect Size
IPS	–	Pre	1.062 (0.431)	0.140 (0.247)	[0.04, 1.09]	0.024	Cohen's d_z	0.57
		Post	1.201 (0.314)					
A_{LOS}	–	Pre	45.900 (30.008)	9.394 (18.558)	[-0.01, 1.02]	0.041	Cohen's d_z	0.51
		Post	55.295 (33.046)					
S_{rect}	Center	Pre	2.856 (2.360)	0.288 (1.578)	[-0.21, 0.66]	0.304	Rank-biserial correlation	0.29
		Post	3.144 (2.573)					
	Front-Right	Pre	4.295 (3.154)	1.160 (2.876)	[-0.22, 0.64]	0.332	Rank-biserial correlation	0.26
		Post	5.455 (4.416)					
	Front	Pre	4.066 (3.390)	-0.992 (2.626)	[-0.72, 0.15]	0.207	Rank-biserial correlation	-0.36
		Post	3.073 (1.781)					
	Front-Left	Pre	3.945 (2.504)	0.723 (1.361)	[-0.00, 1.06]	0.038	Cohen's d_z	0.53
		Post	4.667 (2.730)					
	Left	Pre	4.549 (1.996)	-0.694 (2.197)	[-0.84, 0.21]	0.211	Cohen's d_z	-0.32
		Post	3.854 (2.036)					
	Back-Left	Pre	4.217 (2.771)	1.800 (3.580)	[-0.02, 0.75]	0.096	Rank-biserial correlation	0.44
		Post	6.018 (4.843)					
	Back	Pre	4.505 (2.568)	-0.336 (2.507)	[-0.63, 0.37]	0.578	Cohen's d_z	-0.13
		Post	4.170 (2.461)					
	Back-Right	Pre	4.197 (3.180)	-0.187 (2.685)	[-0.47, 0.50]	0.963	Rank-biserial correlation	0.02
		Post	4.010 (2.887)					
	Right	Pre	5.485 (4.299)	-0.048 (4.447)	[-0.45, 0.48]	0.966	Rank-biserial correlation	0.02
		Post	5.437 (3.385)					

Table S5. Detailed statistical results for the mature cohort (increased group) in the immediate effect verification experiment, including p-values and effect sizes

Index	Direction	Timing	Mean (SD)	Difference Mean (SD)	95% CI	p	Effect Size Type	Effect Size
IPS	–	Pre	0.945 (0.473)	0.236 (0.250)	[0.12, 1.76]	0.011	Cohen's d_z	0.94
		Post	1.181 (0.353)					
A_{LOS}	–	Pre	37.807 (29.102)	20.488 (16.329)	[0.34, 2.17]	0.002	Cohen's d_z	1.25
		Post	58.295 (36.457)					
S_{rect}	Center	Pre	1.821 (0.980)	0.570 (1.490)	[-0.43, 0.77]	0.492	Rank-biserial correlation	0.27
		Post	2.392 (1.848)					
	Front-Right	Pre	4.029 (2.870)	2.379 (3.186)	[-0.02, 1.51]	0.033	Cohen's d_z	0.75
		Post	6.408 (4.746)					
	Front	Pre	3.128 (2.048)	-0.677 (1.726)	[-1.26, 0.48]	0.304	Cohen's d_z	-0.39
		Post	2.451 (0.476)					
	Front-Left	Pre	3.147 (2.328)	1.113 (0.967)	[0.60, 0.97]	0.010	Rank-biserial correlation	0.89
		Post	4.260 (2.211)					
	Left	Pre	4.275 (2.165)	0.000 (2.312)	[-0.77, 0.77]	1.000	Cohen's d_z	0.00
		Post	4.275 (2.289)					
	Back-Left	Pre	3.154 (1.706)	2.374 (3.757)	[-0.05, 0.89]	0.105	Rank-biserial correlation	0.60
		Post	5.528 (4.319)					
	Back	Pre	3.788 (1.512)	0.119 (2.786)	[-0.73, 0.81]	0.901	Cohen's d_z	0.04
		Post	3.907 (2.640)					
	Back-Right	Pre	5.114 (4.066)	0.017 (1.833)	[-0.60, 0.60]	1.000	Rank-biserial correlation	0.00
		Post	5.132 (4.030)					
	Right	Pre	5.726 (3.375)	0.869 (4.211)	[-0.52, 0.93]	0.530	Cohen's d_z	0.21
		Post	6.596 (3.540)					

Table S6. Detailed statistical results for the mature cohort (decreased group) in the immediate effect verification experiment, including p-values and effect sizes

Index	Direction	Timing	Mean (SD)	Difference Mean (SD)	95% CI	p	Effect Size Type	Effect Size
IPS	–	Pre	1.222 (0.328)	0.008 (0.183)	[-0.79, 0.88]	0.902	Cohen's d_z	0.05
		Post	1.230 (0.274)					
A_{LOS}	–	Pre	57.029 (29.346)	-5.859 (6.737)	[-1.87, 0.13]	0.043	Cohen's d_z	-0.87
		Post	51.170 (29.594)					
S_{rect}	Center	Pre	4.639 (3.786)	-1.497 (3.886)	[-0.73, 0.68]	0.945	Rank-biserial correlation	-0.06
		Post	3.141 (2.708)					
	Front-Right	Pre	4.661 (3.680)	-0.516 (1.110)	[-1.35, 0.42]	0.230	Cohen's d_z	-0.46
		Post	4.146 (3.819)					
	Front	Pre	4.417 (4.043)	-1.550 (3.027)	[-0.93, 0.32]	0.219	Rank-biserial correlation	-0.57
		Post	2.867 (2.039)					
	Front-Left	Pre	4.629 (2.517)	-0.203 (1.219)	[-1.10, 0.77]	0.675	Cohen's d_z	-0.17
		Post	4.426 (2.809)					
	Left	Pre	4.856 (1.882)	-1.476 (1.899)	[-1.75, 0.19]	0.064	Cohen's d_z	-0.78
		Post	3.381 (1.733)					
	Back-Left	Pre	4.880 (3.136)	0.810 (3.539)	[-0.62, 1.08]	0.538	Cohen's d_z	0.23
		Post	5.690 (5.272)					
	Back	Pre	4.574 (2.787)	-0.207 (1.537)	[-0.97, 0.71]	0.715	Cohen's d_z	-0.13
		Post	4.367 (2.555)					
	Back-Right	Pre	3.947 (3.065)	-0.478 (3.669)	[-0.72, 0.78]	0.938	Rank-biserial correlation	0.07
		Post	3.469 (2.167)					
	Right	Pre	3.535 (3.118)	0.423 (1.368)	[-0.64, 1.26]	0.444	Cohen's d_z	0.31
		Post	3.959 (2.939)					

Table S7. Detailed statistical analysis of training phase indicators for the younger cohort, including p-values and effect sizes

Variable	Range	Group	Mean (SD)	95% CI	p	Effect Size Type	Effect Size
R_{LOS},x	~25%	Increased	0.170 (0.055)	[-0.080, 0.002]	0.048	Rank-biserial r	-0.41
		Decreased	0.209 (0.060)				
	25~75%	Increased	0.294 (0.063)	[-0.074, 0.048]	0.657	Cohen's d	-0.16
		Decreased	0.308 (0.100)				
	75%~	Increased	0.536 (0.105)	[-0.034, 0.139]	0.226	Cohen's d	0.44
		Decreased	0.484 (0.133)				
R_{LOS},y	~25%	Increased	0.151 (0.065)	[-0.040, 0.052]	0.798	Cohen's d	0.09
		Decreased	0.146 (0.063)				
	25~75%	Increased	0.257 (0.077)	[-0.066, 0.082]	0.825	Cohen's d	0.08
		Decreased	0.249 (0.121)				
	75%~	Increased	0.390 (0.166)	[-0.107, 0.119]	0.865	Rank-biserial r	-0.04
		Decreased	0.384 (0.146)				
CROSSx	–	Increased	46.9 (16.7)	[-25.657, 0.657]	0.062	Cohen's d	-0.69
		Decreased	59.5 (19.6)				
CROSSy	–	Increased	39.1 (20.8)	[-20.812, 6.687]	0.302	Cohen's d	-0.37
		Decreased	46.1 (17.1)				

Table S8. Detailed statistical analysis of training phase indicators for the mature cohort, including p-values and effect sizes

Variable	Range	Group	Mean (SD)	95% CI	p	Effect Size Type	Effect Size
$R_{LOS,x}$	~25%	Increased	0.181 (0.077)	[-0.162, 0.017]	0.109	Rank-biserial r	-0.46
		Decreased	0.254 (0.097)				
	25~75%	Increased	0.298 (0.087)	[-0.113, 0.041]	0.340	Cohen's d	-0.44
		Decreased	0.333 (0.072)				
	75%~	Increased	0.521 (0.158)	[-0.020, 0.237]	0.094	Cohen's d	0.78
		Decreased	0.413 (0.108)				
$R_{LOS,y}$	~25%	Increased	0.104 (0.084)	[-0.105, 0.050]	0.464	Cohen's d	-0.34
		Decreased	0.131 (0.074)				
	25~75%	Increased	0.221 (0.148)	[-0.216, 0.099]	0.440	Cohen's d	-0.38
		Decreased	0.280 (0.166)				
	75%~	Increased	0.567 (0.258)	[-0.094, 0.412]	0.177	Rank-biserial r	0.39
		Decreased	0.408 (0.254)				
CROSSx	–	Increased	110.1 (119.4)	[-69.577, 103.509]	0.836	Rank-biserial r	-0.07
		Decreased	93.1 (52.3)				
CROSSy	–	Increased	47.0 (31.4)	[-31.811, 24.811]	0.797	Cohen's d	-0.12
		Decreased	50.5 (26.8)				
